# Antifungal drug miconazole ameliorated memory deficits in a mouse model of LPS-induced memory loss through targeting iNOS

**DOI:** 10.1038/s41419-020-2619-5

**Published:** 2020-08-14

**Authors:** In Jun Yeo, Jaesuk Yun, Dong Ju Son, Sang-Bae Han, Jin Tae Hong

**Affiliations:** College of Pharmacy and Medical Research Center, Chungbuk National University, 194-31 Osongsaengmyeong 1-ro, Osong-eup, Heungdeok-gu, Cheongju, Chungbuk 28160 Republic of Korea

**Keywords:** Target identification, Neuroimmunology

## Abstract

Alzheimer’s disease (AD) is closely related to neuroinflammation, and the increase in inflammatory cytokine generation and inducible nitric oxide synthase (iNOS) expression in the brain of a patient with AD is well known. Excessive cytokines can stimulate iNOS in microglia and astroglia and overproduce nitric oxide, which can be toxic to neurons. The disease–gene–drug network analysis based on the GWAS/OMIM/DEG records showed that miconazole (MCZ) affected AD through interactions with NOS. Inhibiting iNOS can reduce neuroinflammation, thus preventing AD progression. To investigate the prophylactic role of antifungal agent in the AD development, a lipopolysaccharide-induced memory disorder mouse model was used, and cognitive function was assessed by Morris water maze test and passive avoidance test. MCZ treatment significantly attenuated cognitive impairment, suppressed iNOS and cyclooxygenase-2 expression, and activation of astrocyte and microglial BV2 cells, as well as reduced cytokine levels in the brains and lipopolysaccharide-treated astrocytes and microglia BV2 cells. In further mechanism studies, Pull-down assay and iNOS luciferase activity data showed that MCZ binds to iNOS and inhibited transcriptional activity. Our results suggest that MCZ is useful for ameliorating the neuroinflammation-mediated AD progression by blocking iNOS expression.

## Introduction

Dementia, an age-related disease, is characterized by difficulties with memory, speaking, problem-solving, and other cognitive abilities^1^. There are many types of dementia, including Alzheimer’s disease (AD), Lewy body dementia, mixed dementia, and vascular dementia^1,2^. As the lifespan increases, the number of patients with dementia continues to increase, but the currently used acetylcholine esterase-inhibiting drugs only delay the symptomatic progression, and there is no curative treatment^3^. Other therapeutic agents against amyloid-beta or phospho-tau have been developed as target drugs; however, clinical trials have not been successful^4^. Therefore, it is necessary to develop therapeutic drugs with a new direction.

It is well known that cytokines, chemokines, caspases, nitric oxide (NO), and reactive oxygen species (ROS) cause neuroinflammation and can lead to neurodegenerative diseases^5^. There has been much research illustrating the relationship of iNOS expression and NO generation with neuroinflammation and dementia^6,7^. Previous research has reported a significant rise in NOS activity in the brain microvessels of patients with AD and increased iNOS mRNA in the cortices of patients with AD^8,9^. In an animal model, iNOS knockout mice showed improved cognitive ability and multiple pathologies^10,11^. During the course of inflammation, excessive cytokines can stimulate iNOS in microglia and astroglia cells and overproduce nitric oxide, which can lead to neurodegeneration^12,13^ Nuclear factor-kappa B (NF-κB) is a transcription factor that influences the levels of several inflammatory genes, such as iNOS and COX-2, as well as cytokines^14^. Thus, inhibiting the iNOS pathway directly or indirectly can reduce neuroinflammation and be a good target for drugs as a dementia treatment^15^. To find a new therapeutic agent, we used bioinformatics based on big data to search for any drug that could be useful for dementia treatment^16^. Consequently, we found an interesting drug, miconazole (MCZ), which could be cross linked to iNOS as a dementia target.

Azole drugs have generally been used as antifungal agents, and their main action is to inhibit fungal cell membrane synthesis^17^. Interestingly, these drugs reportedly inhibit iNOS expression^18,19^. There have been reports showing that the neuroprotective effects of MCZ, such as remyelination^20^, protect blood vessels from rupturing and inflammation in hemorrhagic stroke^21^, and it has been shown that MCZ pass the blood–brain barrier following intraperitoneal administration in mice^22^. Lipopolysaccharide (LPS) are molecules typically used to induce iNOS signaling; LPS exposure can therefore increase iNOS expression and neuroinflammation in neuronal cells^23–25^. Thus, an animal study, an intraperitoneal injection of LPS has been used to induce a cognitive impairment model in mice^26–29^.

To investigate whether azole drugs can be useful for AD through the inhibition of iNOS expression and the consequential neuroinflammation, we administered MCZ, an azole drug, and evaluated the anti-AD effect and its active mechanism based on bioinformatics analysis (Supplementary Fig. 1).

## Materials and methods

### Animal experiments

A total of 40 adult 8-week-old male C57BL6/N mice (20–25 g) were purchased from Daehan Biolink (Chungcheongbuk-do, Korea), and were maintained in accordance with the guidelines of the National Institute of Toxicological Research and Korea Food and Drug Administration for the humane care and use of laboratory animals. All experimental procedures in the present study were approved by the IACUC of Chungbuk National University (approval number: CBNUA-1088-18-01). Animals were housed in a room that was automatically maintained at 21–25 °C, with a relative humidity of 45–65% and controlled 12-h light-dark cycle. To induce memory impairment, the mice were randomly allocated to four groups. Group 1 (control group; *n* = 8) received saline vehicles, and group 2 (MCZ group; *n* = 8) received MCZ (40 mg/kg), group 3 (LPS group; *n* = 12) received LPS (250 μg/kg/day), group 4 (MCZ + LPS group; *n* = 12) received both. All injections were given for 7 days with intraperitoneal administration. The concentration of the drug and the route of administration were referred to other research papers, which shows brain MCZ concentration by i.p. injection^22,26^.

### Materials

Miconazole, Fluconazole, and Clotrimazole United States Pharmacopeia (USP) Reference Standard and Lipopolysaccharides from Escherichia coli O111:B4 and other chemicals were obtained from Sigma-Aldrich (USA).

### Morris water maze

The Morris water maze test is a widely accepted method for examining cognitive function and was used in the present study as described previously^30^. Briefly, a circular plastic pool (height 35 cm, diameter 100 cm) was filled with water (plus white dye) maintained at 22–25 °C. An escape platform (height 14.5 cm, diameter 4.5 cm) was submerged 1–1.5 cm below the surface of the water. The test was performed three times a day for 5 days during the acquisition phase (Days 1–5), with three randomized starting points. The position of the escape platform was kept constant. Each trial lasted for 60 s or ended as soon as the mice reached the submerged platform. Swimming pattern of each mouse was monitored and recorded by a camera mounted above the center of the pool, and the escape latency, escape distance and swimming speed were assessed by the SMART-LD program (Panlab, Spain). A quiet environment, constant water temperature was maintained throughout the experimental period.

### Probe test

To assess memory consolidation, a probe test was performed 24 h after the water maze test (i.e., Day 6). For the probe test, the platform was removed from the pool and the mice could swim freely. The swimming pattern of each mouse was monitored and recorded for 60 s using the SMART-LD program (Panlab). Consolidated spatial memory was estimated by the time spent in the target quadrant area.

### Passive avoidance performance test

The passive avoidance response was determined using a “step-through” apparatus (Med Associates, USA) that is divided into an illuminated compartment and a dark compartment (each 20.3 × 15.9 × 21.3 cm) adjoining each other through a small gate with a grid floor, 3.175 mm stainless steel rods set 8 mm apart. Twenty-four after the probe test (i.e., Day 7) 3 min habituation in gate opened chamber with no shock. Forty-eight hours after the probe test (i.e., Day 8), a training trial was performed. The mice were placed in the illuminated compartment facing away from the dark compartment for the training trial. When the mice moved completely into the dark compartment, it received an electric shock (0.4 mA, 3 s duration). Then, the mice were returned to their cage. Twenty-four hours after the training trial (i.e., Day 9), each mouse was placed in the illuminated compartment and the latency period to enter the dark compartment defined as “retention” was measured. The time when the mice entered into the dark compartment was recorded and described as step-through latency. The retention trials were set at a cutoff time limit of 180 s.

### Brain collection and preservation

After behavioral tests, mice were perfused with phosphate-buffered saline (PBS, pH 7.4) with heparin under inhaled CO_2_ anesthetization. The brains were immediately removed from the skulls and divided into left brain and right brain. One stored at −80 °C, the other were fixed in 4% paraformaldehyde for 72 h at 4 °C and transferred to 30% sucrose solutions.

### Open-field test

Locomotor activity was tested in a 61 × 61 cm open-field arena (*n* = 6 per group). Mice were independently placed in one corner of the arena and horizontal movements were recorded for 30 min with Any-Maze Behavior Video Tracking Software SMART-LD program (Panlab). The open-field chamber was cleaned between trials with 70% v/v alcohol solution.

### Aβ_1–42_-induced animal experiment

Eight-to-10-week-old male C57BL6/N mice (Daehan Biolink, Chungcheongbuk-do, Republic of Korea) were maintained and handled in accordance with the humane animal care and use guidelines of Korean FDA. The infusion mouse model was adapted from previous work on the rat infusion model. To induce memory impairment, the mice were randomly allocated to three groups. Group 1 (control group; *n* = 10) received saline vehicles 7 days after surgery, and group 2 (Aβ infusion; *n* = 10) received saline vehicles 7 days after surgery, group 3 (Aβ infusion; *n* = 10) received MCZ (40 mg/kg/day) 7 days after surgery. The anesthetized animals were placed in a stereotaxic instrument, and catheters were attached to an osmotic mini-pump (Alzet 1002, ALZA, Mountain View, CA, USA) and a brain infusion kit 1 (Alzet kit 3–5 mm, ALZA) that were implanted according to the following coordinates: mouse (unilaterally): −1.0 mm anterior/posterior, +0.5 mm medial/lateral, and −2.5 mm dorsal/ventral. The pump contents were released over a period of 14 days consisting of 300 pmol aggregated Aβ1–42 (Bachem Chemical, Torrance, CA, USA) dissolved in a sterile saline (0.9% NaCl) for each pump. The behavioral tests of learning and memory capacity were then assessed using three tests (water maze, probe, and passive avoidance tests).

### Astrocytes and microglial BV2 cells culture

Astrocytes were prepared from the cerebral cortex of rat embryos (E18). After the skull was cut and the skin was opened, the brain was released from the skull cavity. After washing with PBS, the cerebrum was separated from the cerebellum and brain stem, and the cerebral hemispheres were separated from each other by gently teasing along the midline fissure with the sharp edge of forceps. The meninges were gently peeled from the individual cortical lobes and the cortices were dissociated by mechanical digestion [using the cell strainer (BD Bioscience, Franklin Lakes, NJ, USA)]. The resulting cells were centrifuged (1,500 rpm, 5 min), resuspended in serum-supplemented culture media, and plated into 100 mm dishes. The cells were seeded on culture 100 mm dishes and incubated in Dulbecco’s modified eagle medium (DMEM)/F-12 (Invitrogen, Carlsbad, CA) containing 10% fetal bovine serum (FBS) (Invitrogen). The culture medium was replaced every 3 days thereafter. After 14 days, the cultures became confluent and loosely attached microglia and oligodendrocyte precursor cells were removed from the cell monolayer using shaking incubator (37 °C, 350 RPM, 2–4 h). Astrocytes were subsequently detached using trypsin-EDTA and plated into 100 mm cell culture dishes. As described elsewhere^26^, the percentage of astrocytes in our culture system is >95%. The murine BV2 cell line (a generous gift from W. Kim, KRIBB (Korea Research Institute of Bioecience & Biotechnology), Daejeon, Korea), which becomes immortal after infection with a v-raf/v-myc recombinant retrovirus, exhibits phenotypic and functional properties of reactive microglial cells. BV2 cells were maintained at 37 °C and 5% CO2 in Dulbecco’s Modified Eagle Medium (DMEM) supplemented with 10% heat-inactivated endotoxin-free FBS, 2 mM glutamine, 100 µg/mL streptomycin and 100 µg/mL penicillin. BV2 cells were grown in 6-well plates at a concentration of 2 × 10^5^ cells/well, followed by proper treatment.

### Immunohistochemical staining

After being transferred to 30% sucrose solutions, brains were embedded into paraffin wax, routinely processed and then sectioned into 10 μm thick slices by using rotary microtome (Leica RM 2125 RTS, Singapore). The sections were bionylated with GFAP (Santa cruz, #sc-33673), Iba-1 (Wako, #019-19741), iNOS (Thermo, #PA3-030A), COX-2 (abcam, #ab52237) antibody (1:100) overnight incubation at 4 °C, and anti-IgG-horseradish peroxidase (HRP) secondary antibodies (1:500; Santa Cruz Biotechnology, Inc., Santa Cruz, CA, USA), 1 h 30 min incubation at room temperature. Brain sections visualized by a chromogen DAB (Vector Laboratories) reaction for up to 10 min and counterstained with hematoxylin for 30 s. Finally, brain sections were dehydrated in ethanol, cleared in xylene, mounted with Permount (Fisher Scientific, Hampton, NH), and evaluated on a light microscopy (Microscope Axio Imager.A2, Carl Zeiss, Oberkochen, Germany) (×50 and × 200).

### Western blotting

In in vivo study, for comparing the expression of protein levels through western blotting, we selected and used 3 of 12 mice brain from each group. Protein was extract by PRO-PREP™ Protein Extraction Solution (iNtRON Biotechnology, Inc., Seongnam, Korea). An equal amount of total protein (10 μg) was resolved on 8–15% sodium dodecyl sulfate polyacrylamide gel (SDS-PAGE) and then transferred to a nitrocellulose membrane (Hybond ECL; Amersham Pharmacia Biotech, Piscataway, NJ, USA). The membranes were blocked for 1 h in 2.5% skim milk solution and incubated overnight at 4 °C with specific antibodies. To detect target proteins, specific antibodies against iNOS (abcam, #3523), COX-2 (Novus, #NB110-1948), Iba-1 (Novus, #NBP2-16908), p65 (Santa cruz, #sc-8008), phosphor-IκB (CST, #9246S), IκB (CST, #9242 S), GFAP (Santa cruz, #sc-33673), p50 (Santa cruz, #sc-8414), Histone H3 (Santa cruz, #sc-8654), β-actin (Santa cruz, #sc-47778) were used (1:1000), overnight incubation at 4 °C. The blots were then incubated with the corresponding conjugated goat anti-rabbit or goat anti-mouse or donkey anti-goat IgG-horseradish peroxidase (HRP) (1:5000; Santa Cruz Biotechnology Inc. Santa Cruz, CA, USA) secondary antibodies, 1 h 30 min incubation at room temperature. Immunoreactive proteins were detected with an enhanced chemiluminescence western blotting detection system. The relative density of the protein bands was scanned by densitometry using MyImage (SLB, Seoul, Korea).

### RNA isolation and quantitative real-time reverse transcriptase polymerase chain reaction (RT-PCR)

Tissue RNA was isolated from homogenized hippocampus using RiboEX (Gene All, Seoul, Korea), and total RNA (0.2 μg) was reverse transcribed into complementary DNA (cDNA) according to the manufacturer’s instructions using Applied Biosystems (Foster City, CA, USA). For the quantitative, real-time, reverse transcriptase polymerase chain reaction (PCR) assays, the linearity of the amplifications of iNOS, tumor necrosis factor (TNF)-α, interleukin (IL)-1β, IL-6, and GAPDH cDNAs was established in preliminary experiments. All signal mRNAs were normalized to GAPDH mRNA. cDNAs were amplified by real-time PCR in duplicate with QuantiNova SYBR green PCR kit (Qiagen, Valencia, CA, USA). Each sample was run with the following primer sets: miNOS, 5ʹ-TGACGCTCGGAACTGTAGCAC-3ʹ (sense), 5ʹ-TGATGGCCGACCTGATGTT-3ʹ (antisense); mTNF-α, 5ʹ-TGTAGCCCACGTCGTAGCAA-3ʹ (sense), 5ʹ-AGGTACAACCCATCGGCTGG-3ʹ (antisense); mIL-1β, 5ʹ-TGCCACCTTTTGACAGTGATG-3ʹ (sense), 5ʹ-ATGTGCTGCTGCGAGATTTG-3ʹ (antisense); mIL-6, 5ʹ-CCACTTCACAAGTCGGAGGC-3ʹ (sense), 5ʹ-GCCATTGCACAACTCTTTTCTCA-3ʹ (antisense); mGAPDH: 5ʹ-AGGTCGGTGTGAACGGATTTG-3ʹ (sense), 5ʹ-TGTAGACCATGTAGTTGAGGTCA-3ʹ (antisense).

### Cytokine assay

Tissue levels of mouse TNF-α, IL-6, and IL-1β were measured by enzyme-linked immunosorbent assay (ELISA) kits provided by R&D systems (Minneapolis, MN, USA) according to the manufacturer’s protocol.

### Nitrite assay

Astrocytes and microglial BV2 cells were plated at a density of 3 × 10^5^ cells/well in 6-well plates per 2 mL medium for 24 h. After removing the culture medium, the cells were then treated with LPS (1 μg/mL) and miconazole (1.25, 2.5, 5, 10 μM) per 2 mL medium for 24 h. The nitrite in the supernatant was assessed using a NO detection kit (iNtRON Biotechnology, Seongnam, Korea), according to the manufacturer’s instructions. Finally, the resulting color was assayed at 520 nm using a microplate absorbance reader (VersaMax ELISA, Molecular Devices, California, USA).

### Plasmid vector and luciferase activity assay

BV2 cells were plated in 12-well plates (1 × 10^5^ cells/well) and were transiently transfected with pNF-κB-Luc plasmid was used to assess the NF-kB activity (5x NF-κB; Stratagene, La Jolla, CA), iNOS promoter dual-reporter (MPRM38938-LVPG04) and negative control (NEG-LVPG04) lentiviral plasmid vectors purchased from GeneCopoeia (Rockville, MD, USA), using a mixture of plasmid (20 nM) and the Lipofectamine 3000 reagent in OPTI-MEM, according to the manufacturer’s specification (Invitrogen). The transfected cells were treated with LPS (1 μg/mL) and different concentrations (1.25, 2.5, 5, 10 μM/mL) of miconazole for 12 h. Luciferase activity was measured by using the Secrete-Pair™ Dual Luminescence Assay kit (Genecopoeia, Rockville, MD, USA), and reading the results on a luminometer as described by the manufacturer’s specifications (WinGlow, Bad Wildbad, Germany).

### Pull-down assay

BV2 cell lysate was conjugated with epoxy-activated sepharose 6B (Sigma-Aldrich, St. Louis, MO). iNOS (1 mg) was dissolved in 1 mL of coupling buffer (35% DMSO and 0.5 M NaCl, pH 8.3). The epoxy-activated sepharose 6B was swelled and washed in 1 mM HCl through a sintered glass filter, then washed with a coupling buffer. Epoxy-activated sepharose 6B beads were added to the MCZ-containing coupling buffer and incubated at 4 °C for 24 h. The iNOS-conjugated sepharose 6B was washed with three cycles of alternating pH wash buffers (buffer 1, 0.1 M acetate and 0.5 M NaCl, pH 4.0; buffer 2, 0.1 M Tris-HCl and 0.5 M NaCl, pH 8.0). iNOS-conjugated beads were then equilibrated with a binding buffer (0.05 M Tris-HCl and 0.15 M NaCl, pH 7.5). The control unconjugated epoxy-activated sepharose 6B beads were prepared as described above in the absence of iNOS. The cell lysate was mixed with iNOS-conjugated sepharose 6B or sepharose 6B at 4 °C for 24 h. The beads were then washed three times with Tris-buffered saline and Tween 20 (TBST). The bound proteins were eluted with SDS loading buffer. The proteins were then resolved by SDS-PAGE followed by immunoblotting with antibodies against iNOS (1:1000, Novus Biologicals, Inc., Littleton).

### Docking procedure

Docking studies between MCZ and iNOS were performed using Autodock VINA. Three-dimensional structures of the iNOS-7-nitrodazole bound complexes were retrieved from the Protein Data Bank [PDB:1M8E], and a three-dimensional structure of iNOS was built using Chem3D and ChemDraw, which was further prepared using AutodockTools. The grid box was centered on the iNOS monomer, and the size of the grid box was adjusted to include the whole monomer. Molecular graphics for the best binding model were generated using the Discovery Studio Visualizer.

### Fluorescence microscopy

The fixed cells were exposed to the following primary antibodies: p65 (1:100, Santa Cruz Biotechnology Inc. Santa Cruz, CA, USA), at room temperature for 2 h. After incubation, the cells were washed twice with ice-cold PBS and incubated with an anti-mouse secondary antibody conjugated to Alexa Fluor 568 nm (Invitrogen-Molecular Probes, Carlsbad, CA) at room temperature for 1 h. Immunofluorescence images were acquired using an K1-Fluo laser scanning confocal microscope (Nanoscope Systems, Daejeon, Korea).

### Cell viability assay

Each cell line (microglial BV2 and cultured astrocytes 1 × 10^4^ cells) was cultured for 24 h and then as treated with LPS (1 μg/mL) and/or miconazole (1.25, 2.5, 5, 10, 20 μM) for 24 h. After incubation for 24 h at 37 °C, MTT (3-(4,5-dimethylthiazol-2-yl)−2,5-diphenyltetrazolium bromide; Sigma, St. Louis, MO, USA) diluted in DMEM medium, were added to each well and incubation was carried out for 90 min. Then, the supernatant was discarded, and the crystal products were eluted with DMSO (200 μL/well; Sigma, St. Louis, MO, USA). Colorimetric evaluation was performed with a spectrophotometer at 540 nm to detect cell growth.

### Bioinformatics

Use open source Open Targets Platform (www.targetvalidation.org) and Disease-connect (disease-connect.org).

### Statistical analysis

Data were analyzed by using GraphPad Prism v5.0. All data was examined for normal distribution with D’Agostino & Pearson normality test. If data set exhibited normal distribution, unpaired two-tailed student’s *t*-test or two-way ANOVA for repeated measures and Bonferroni post-hoc analysis was used. If data set did not show normal distribution, two-tailed Mann–Whitney *U-*test was used. To ensure proper animal model, initial experience (random number table method) was assigned to each group according to previous experience (considering mouse mortality and successful model establishment). The data did not include people, so we did not use blinds for this experiment. Data are represented as the mean ± S.E.M. All experiments were performed in triplicate.

## Results

### Miconazole improves memory impairment in LPS-treated mice

**Fig. 1:**
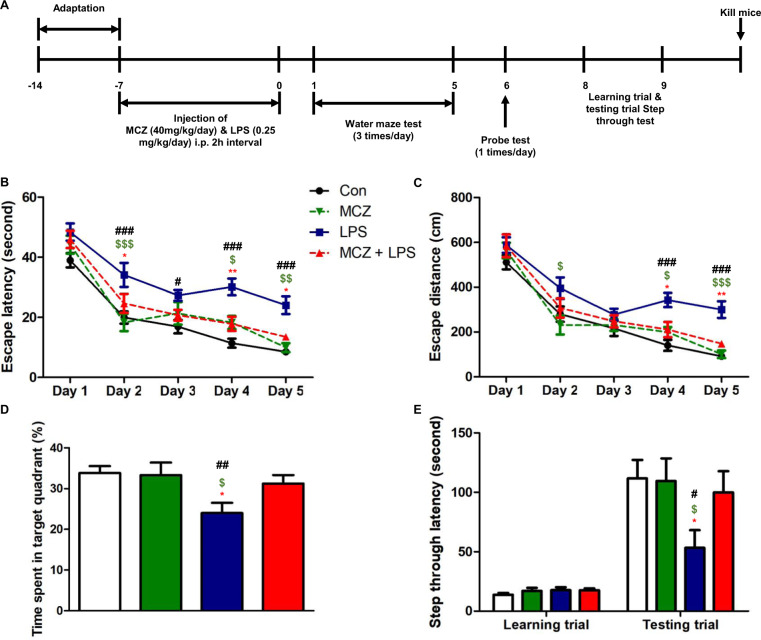
Miconazole attenuates memory impairment in LPS-treated mice. The timeline depicts MCZ treatment and assessments of cognitive functions of mice (**a**). MCZ was injected intraperitoneally and LPS or saline was injected 2 h later MCZ injection, this schedule progressed daily for 7 days. After 7 days of injection, memory tests were conducted; the Morris water maze test was performed three times a day for 5 days. Escape latency (**b**) and distance (**c**) to arrive at the platform were automatically recorded. Data are expressed as the mean ± SEM (Two-way ANOVA for repeated measures followed by post-hoc Bonferroni, ^#^*p* < 0.05, ^##^*p* < 0.01, ^###^*p* < 0.0001 vs. Con, ^$^*p* < 0.05, ^$$^*p* < 0.01, ^$$$^*p* < 0.0001 vs. MCZ, *p < 0.05, **p < 0.01, ***p < 0.0001 vs. MCZ + LPS). After the Morris water maze test, a probe test was performed. The time spent in the target quadrant and target site crossing was within 60 s (**d**). Data are expressed as the mean ± SEM the passive avoidance test was performed 1 day after a learning trial test (**e**). The total number of mice were 40 (control group; *n* = 8, MCZ group; *n* = 8, LPS group; *n* = 12 and MCZ + LPS group; *n* = 12). The mice were given an electric shock when they entered the dark compartment. Data are expressed as the mean ± SEM (*t-*test, ^#^*p* < 0.05, ^##^*p* < 0.01, ^###^*p* < 0.0001 vs. Con, ^$^*p* < 0.05, ^$$^*p* < 0.01, ^$$$^*p* < 0.0001 vs. MCZ, *p < 0.05, **p < 0.01, ***p < 0.0001 vs. MCZ + LPS).

Our results showed that there was some weight loss in early injection days, however, at day 8 they recovered to normal weight that showed there was no significant difference between control group (Supplementary Fig. 2A); and open-field test result demonstrated daily low-dose LPS injection that had no significant effect on sickness behavior (Supplementary Fig. 2B). We first examined the effects of MCZ in an Aβ_1–42_-induced mice memory impairment model. However, our result did not prove significant effect of MCZ in this model (Supplementary Fig. 3)^31^. As MCZ could act as an inflammation inhibitory drug, and LPS-induced AD model could be useful for neuroinflammatory AD model, we examined effect of MCZ on memory improving ability in the LPS-induced AD model. Eventhough, LPS-induced animal model for an AD model have been used in several groups including our team, we first checked the animal status: weight change and sickness behavior. Next, to investigate the spatial learning and memory improvement effects of MCZ on the LPS-induced memory impairment mouse model, the Morris water maze test and passive avoidance test were performed after 7 days of daily injections (Fig. 1a). An intraperitoneal treatment of MCZ (40 mg/kg) daily for 1 week effectively recovered the LPS-induced (250 μg/kg/day) memory loss in mice. Although the dose (40 mg/kg) was much higher than the general antifungal dose (50 mg/day for 14 days), it is reported that the drug could be delivered to the brain effectively by i.p. injection (40 mg/kg; after 6 h 1510.5 ng/g in brain) without any concerns of toxicity in C57BL/6 adult female mice^22^. The group 3 (LPS group) (24.17 ± 2.96 s) (*F* = 83.15, *p-* value < 0.0001) learned slower than the group 1 (Con group) mice (8.5 ± 0.45 s), and group 4 (MCZ + LPS group) (13.47 ± 0.65 s) showed a significant reduction in escape latency on day 5 (Fig. 1b). Group 4 (147.7 ± 10.49 cm) (*F* = 83.44, *p*-value < 0.0001) also showed a shorter escape distance (Fig. 1c) than group 3 (300.2 ± 37.37 cm). The next day, we performed a probe test to check the time spent in the target quadrant zone to test memory maintenance. Group 4 (31.26 ± 2.09%) spent much more time in the quadrant zone than group 3 (24.01 ± 2.53%) (Fig. 1d). Earning the memory capacities was also evaluated by the passive avoidance test. Although the step-through latency did not significantly differ in the learning trial; group 4 (108.7 ± 18.81 s) showed increased step-through latency compared with group 3 (53.32 ± 14.87 s) in the testing trial (Fig. 1e).

### Miconazole reduces neuroinflammation in LPS-treated mice brain

**Fig. 2:**
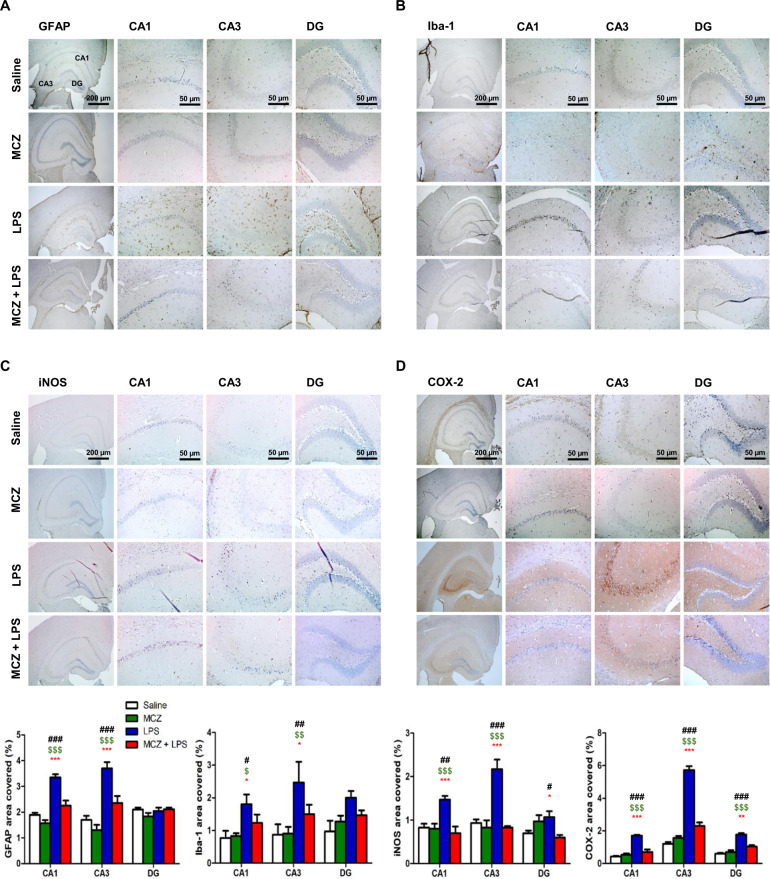
Miconazole reduces neuroinflammation in LPS-induced mice hippocampus. The immunostaining of GFAP (**a**), Iba-1 (**b**), iNOS (**c**), and COX-2 (**d**) proteins in the hippocampus was performed in 10-µm-thick sections of mice brains with specific primary antibodies and biotinylated secondary antibodies. In the upper-right values mean the dyed density in the hippocampus area (DG, CA1, CA3). Quantifications of the areas positively labeled with GFAP, Iba-1, iNOS, or COX-2 were performed using the ImageJ. Quantification results (*n* = 3 mice per group) were analyzed using two-way ANOVA, followed by Bonferroni posttests. Data are expressed as the mean ± SEM (two-way ANOVA for repeated measures followed by post-hoc Bonferroni, ^#^*p* < 0.05 ^##^*p* < 0.01, ^###^*p* < 0.0001 vs. Con, ^$^*p* < 0.05, ^$$^*p* < 0.01, ^$$$^*p* < 0.0001 vs. MCZ, *p < 0.05, **p < 0.01, ***p < 0.0001 vs. MCZ + LPS). Representative images of immunohistochemistry staining with respective antibodies are shown here. Full immunohistochemistry results are included in Supplementary Fig. 4.

**Fig. 3:**
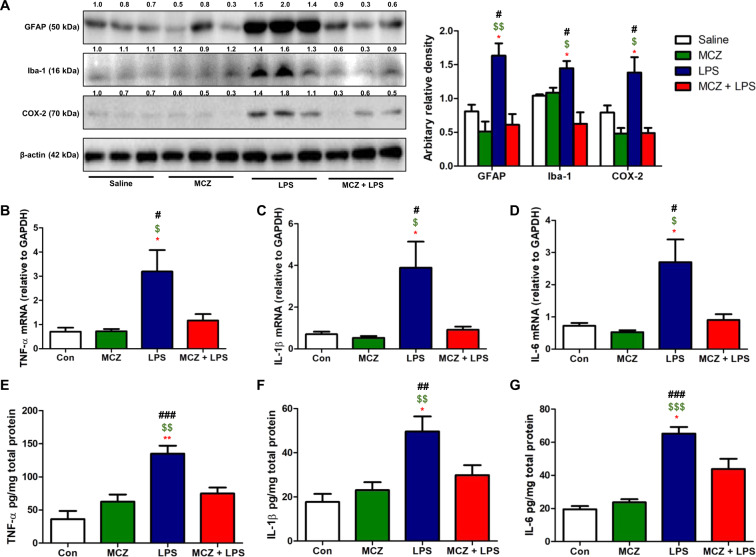
Miconazole reduces neuroinflammation in LPS-treated mice brain. The expression levels of GFAP, Iba-1, and COX-2 in the mice brains were detected by western blotting using specific antibodies. The experiment was performed in triplicate. β-actin levels were measured to confirm equal protein loading. The values on the western blot bands and graph represent the arbitrary density measured by ImageJ (**a**). The mRNA levels of TNF-α (**b**), IL-1β (**c**), and IL-6 (**d**) were detected by qRT-PCR in mice hippocampus. The protein levels of TNF-α (**e**), IL-1β (**f**), and IL-6 (**g**) were detected by ELISA in mice hippocampus. Data are expressed as the mean ± SEM (*t*-test, ^#^*p* < 0.05, ^##^*p* < 0.01, ^###^*p* < 0.0001 vs. Con, ^$^*p* < 0.05, ^$$^*p* < 0.01, ^$$$^*p* < 0.0001 vs. MCZ, *p < 0.05, **p < 0.01, ***p < 0.0001 vs. MCZ + LPS).

Excessive neuroinflammation affects memory loss through the activation of astrocytes and microglia cells. Therefore, we performed immunohistochemistry (IHC) and western blotting to detect the activation of glial cells by GFAP (a marker of astrocytes), Iba-1 (a marker of microglia), and the inflammatory proteins iNOS and COX-2 in the brains. MCZ-treated mice showed fewer GFAP-reactive and Iba-1-reactive cells in the brain than LPS-injected mice (Fig. 2a, b and Supplementary Fig. 4A, B for full analysis). The reactive cells for the inflammatory proteins (iNOS and COX-2) was also lower in MCZ + LPS-treated mice brains than in LPS + saline-treated mice brains (Fig. 2c, d and Supplementary Fig. 5A, B for full analysis). Western blotting also showed that MCZ treatment reduced the expression of GFAP, Iba-1, and COX-2 in hippocampus tissue of mice treated with LPS (Fig. 3a). We found that MCZ treatment decreased the mRNA levels of TNF-α (Fig. 3b), IL-1β (Fig. 3c), and IL-6 (Fig. 3d) in brain hippocampus tissues. We further perform ELISA that MCZ treatment significantly decreased the protein levels of TNF-α (Fig. 3e), IL-1β (Fig. 3f), and IL-6 (Fig. 3g) in brain hippocampus tissues.

### Miconazole prevents LPS-stimulated inflammation in cultured cells

**Fig. 4:**
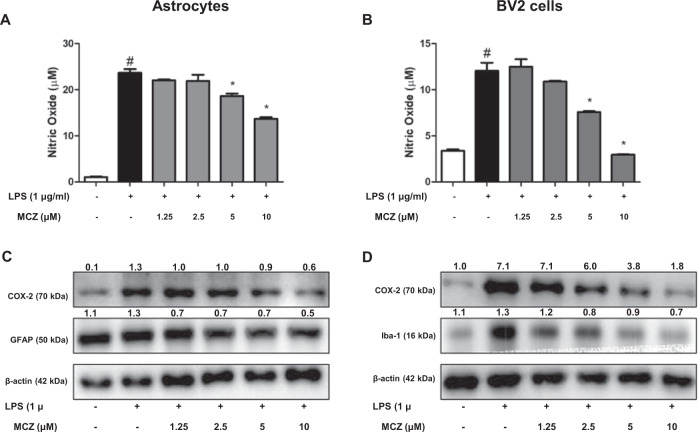
Miconazole effects on NO generation and inflammation in LPS-treated cultured astrocytes and microglial BV2 cells. The NO generation levels were measured in 24-h LPS-treated (1 μg/mL) cultured astrocytes (**a**) and microglial BV2 cells (**b**) pretreated with MCZ (1.25, 2.5, 5, and 10 μM) for 1 h. COX-2, GFAP, and Iba-1 proteins were detected by western blotting using specific antibodies in cultured astrocytes (**c**) and microglial BV2 cells (**d**). β-actin levels were measured to confirm equal protein loading. All experiments were performed three times with duplicate. The values on the western blot bands represent the arbitrary density measured by ImageJ. Significant difference from the control group (*t*-test, *p* < 0.05). ^#^Significant difference from the LPS-treated group (*p* < 0.05). *Significant difference from the MCZ-treated group (*p* < 0.05).

**Fig. 5:**
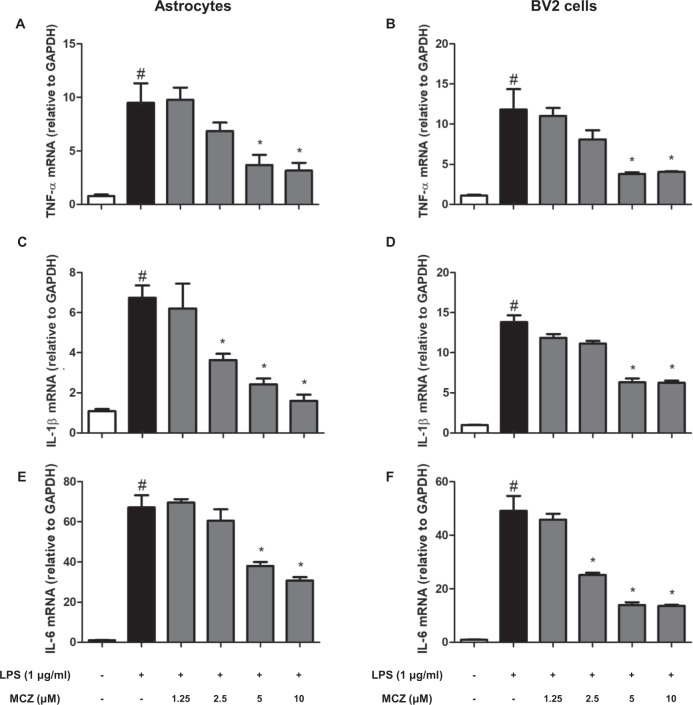
Miconazole effects on pro-inflammatory cytokine production in LPS-treated microglial BV2 cells and cultured astrocytes. The mRNA levels of pro-inflammatory cytokine were measured in 24-h LPS-treated (1 μg/mL) microglial BV2 cells and cultured astrocytes pretreated with MCZ (1.25, 2.5, 5, and 10 μM) for 1 h. The mRNA levels of TNF-α (**a**, **b**), IL-1β (**c**, **d**), and IL-6 (**e**, **f**) were detected by qRT-PCR in microglial BV2 cells (**a**, **c**, **e**) and cultured astrocytes (**b**, **d**, **f**). GAPDH for internal control. All experiments were performed three times with duplicate. Significant difference from the control group (*t*-test, *p* < 0.05). ^#^Significant difference from the LPS-treated group (*p* < 0.05). *Significant difference from the MCZ-treated group (*p* < 0.05).

An in vivo study demonstrated that MCZ attenuated LPS-induced neuroinflammation. We further investigated the anti-inflammatory effects in cultured astrocytes and microglial BV2 cells after LPS treatment (1 µg/mL) with MCZ. The possible concentration range of MCZ was first determined through MTT cell proliferation assay in astrocytes and microglial BV2 cells (Supplementary Fig. 6). Up to 10 µM of MCZ did not show any cytotoxic effect; thus, we used MCZ (1.25, 2.5, 5, and 10 µM) in a further study. NO was first determined because NO could be a significant inflammatory product, and GWAS analysis showed iNOS expression could be significant for the development of AD (Supplementary Fig. 1). It was detected that the NO level was decreased by treatment of MCZ concentration dependently in astrocyte cells (Fig. 4a) and microglial BV2 cells (Fig. 4b). Additionally, we determined whether other azole compounds had a similar effect on NO generation using the NO assay. NO generation levels were also decreased by other azole drugs (fluconazole and clotrimazole) in BV2 cells (Supplementary Fig. 7A). Then, we detected the expression of COX-2 as well as marker proteins of astrocytes (GFAP) and microglial cells (Iba-1) using western blotting. MCZ reduced the LPS-induced increase in the expression of COX-2 in a concentration-dependent manner in cultured astrocyte cells (Fig. 4c) and microglial BV2 cells (Fig. 4d). The mRNA levels of the LPS-induced pro-inflammatory cytokines TNF-α (Fig. 5a, b), IL-1β (Fig. 5c, d), and IL-6 (Fig. 5e, f) were also reduced concentration dependently by MCZ in astrocyte and BV2 cells. We further perform ELISA that MCZ treatment significantly decreased the protein levels of TNF-α (Supplementary Fig. 8A), IL-1β (Supplementary Fig. 8B), and IL-6 (Supplementary Fig. 8C) in BV2 cells supernatant.

### Miconazole binds to iNOS, and then inhibits iNOS expression

**Fig. 6:**
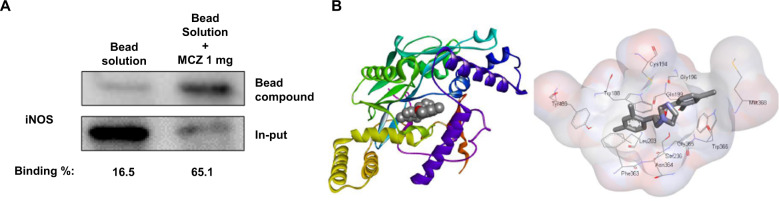
Miconazole-binding affinity to iNOS. Whole-cell lysates of BV2 were incubated with MCZ-conjugated sepharose 6B. After precipitation, the levels of bound iNOS were monitored by western blot analysis (**a**). The under values mean the binding percentage with bead measured by ImageJ. Docking model of MCZ with iNOS (**b**).

In an in vivo and in vitro study, we investigated the significant involvement of iNOS as a target of MCZ. First, the interaction of MCZ-sepharose 6B beads with cell lysate containing iNOS was then detected by immunoblotting with anti-iNOS antibody. The result indicated that MCZ interacted with cell lysates containing iNOS in cultured microglial BV2 cells (Fig. 6a). To identify the binding site of MCZ to iNOS, we performed computational docking experiments with MCZ and iNOS (Fig. 6b). We found that MCZ directly binds iNOS with the strongest protein-binding affinity (−9.6 kcal/mol) to Trp188, Cys194, Gly196, Gln199, Leu203, Ser236, Phe363, Asn364, Gly365, Trp366, Met368, and Tyr483 in the docking model.

**Fig. 7:**
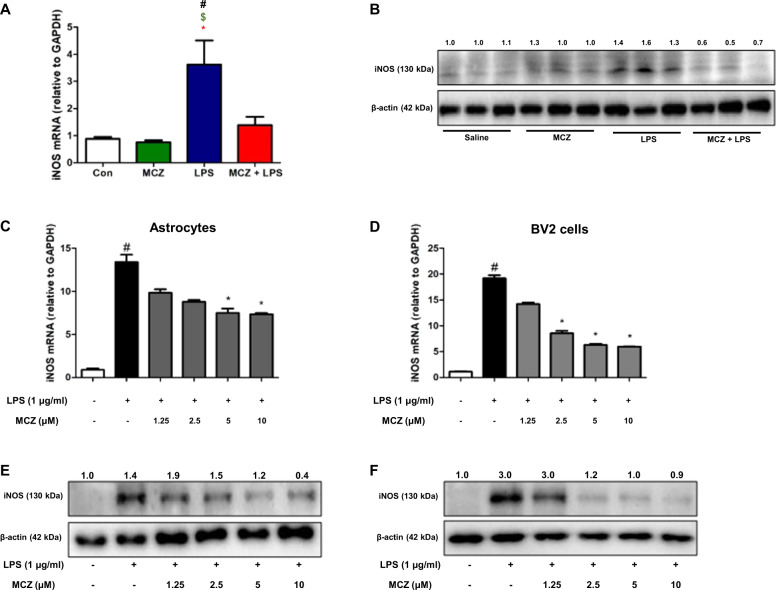
Miconazole inhibits iNOS expression in in vivo and in vitro. The mRNA levels of iNOS were measured in mice hippocampus tissues (**a**) and 24-h LPS-treated (1 μg/mL) cultured astrocytes (**c**) and microglial BV2 cells (**d**) pretreated with MCZ (1.25, 2.5, 5, and 10 μM) for 1 h. iNOS proteins were detected by western blotting using specific antibodies in mice hippocampus tissues (**b**), cultured astrocytes (**e**), and microglial BV2 cells (**f**). β-actin levels were measured to confirm equal protein loading. All experiments were performed three times with duplicate. The values on the western blot bands represent the arbitrary density measured by ImageJ. Data are expressed as the mean ± SEM (*t-*test, ^#^*p* < 0.05 vs. Con, ^$^*p* < 0.05 vs. MCZ, *p < 0.05 vs. MCZ + LPS). Significant difference from the control group (*t*-test, *p* < 0.05). ^#^Significant difference from the LPS-treated group (*p* < 0.05). *Significant difference from the MCZ-treated group (*p* < 0.05).

We also found that the mRNA level of iNOS was decreased in mouse hippocampus (Fig. 7a) and concentration dependently in astrocyte cells (Fig. 7c) and microglial BV2 cells (Fig. 7d). Additionally, we determined whether other azole compounds had a similar effect on iNOS mRNA and NO generation using the NO assay and by analyzing the mRNA levels using qPCR. NO generation and iNOS mRNA levels were also decreased by other azole drugs (fluconazole and clotrimazole) in BV2 cells. These azole drugs had a significant effect on NO generation (Supplementary Fig. 7A) and iNOS mRNA levels (Supplementary Fig. 7B). Then, we detected the expression of iNOS using western blotting. MCZ reduced the LPS-induced increase in the expression of iNOS in a mice hippocampus tissues (Fig. 7b) and concentration-dependent manner in microglial cultured astrocyte cells (Fig. 7e) and BV2 cells (Fig. 7f). In addition, whether MCZ and other azole compounds (fluconazole and clotrimazole) could influence iNOS expression, we induced luciferase activity in microglia BV2 cells. MCZ significantly and most effectively concentration dependently inhibited LPS-induced iNOS luciferase activity was used to assess the iNOS expression (Supplementary Fig. 7C).

### Miconazole prevents LPS-stimulated nuclear translocation of the NF-κB subunit protein through inhibiting IκB phosphorylation

**Fig. 8:**
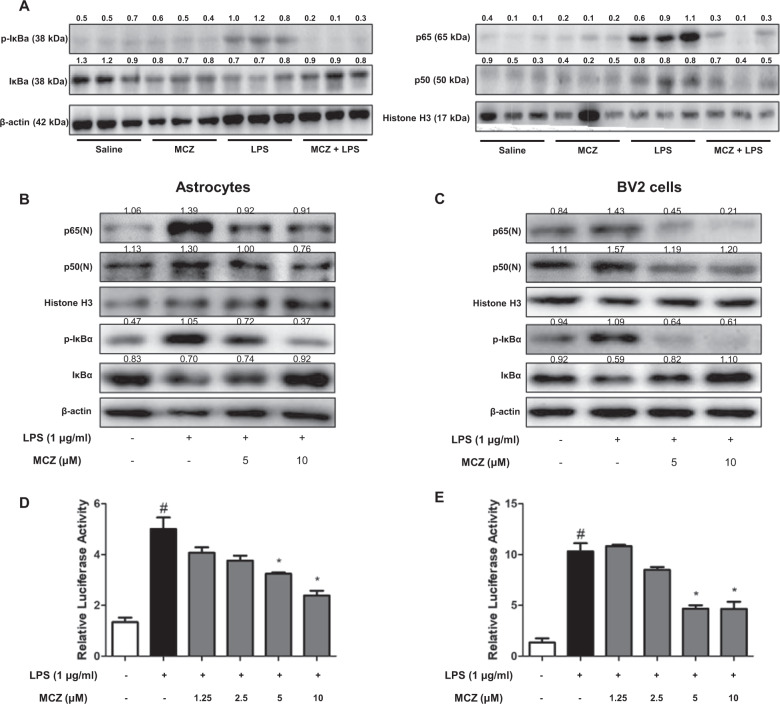
Miconazole effects on activity of NF-κB in LPS-treated mice brain and LPS-treated microglial BV2 cells and cultured astrocytes. To observe the activity of NF-κB, the expressions of NF-κB-related proteins, such as IκB, p-IκB, p65, and p50 were detected by western blotting using specific antibodies in the mouse brains (**a**). The activity of NF-κB was measured in 30-m LPS-treated (1 μg/mL) microglial BV2 cells and cultured astrocytes pretreated with MCZ (5 μM and 10 μM) for 1 h. The expressions of proteins, such as IκB, p-IκB, p65, and p50, were detected by western blotting using specific antibodies in the microglial BV2 cells (**b**) and cultured astrocytes (**c**). Each blot was representative of three experiments. N: nuclear. The values on the western blot bands represent the arbitrary density measured by ImageJ. The effects of MCZ on LPS-induced NF-κB-dependent luciferase activity in microglial BV2 cells and cultured astrocytes. Microglial BV2 cells (**d**) and cultured astrocytes (**e**) were transfected with pNF-κB–Luc plasmid (5 × NF-κB) and then activated with LPS in the absence or presence of MCZ for 12 h, and then, the luciferase activity was determined. Values are the mean and SEM of three independent experiments performed in triplicate. The induction level was calculated relative to the luciferase activity in unstimulated transfected cells. All experiments were performed three times with duplicate. ^#^Significant difference from the LPS-treated group (*p* < 0.05). *Significant difference from the MCZ-treated group (*p* < 0.05).

NF-κB, as a transcriptional factor regulating iNOS, is critical for neuroinflammation and iNOS expression. We examined the protein expression of the functional subunits of the NF-κB complex; p50 and p65 using western blotting. For the nuclear translocation of the NF-κB complex, IκB must be phosphorylated, and then p65 and p50 translocate to the nucleus. It was confirmed that MCZ decreased the expression of phosphorylated IκB and translocation of p50 and p65 in the mice hippocampus tissues (Fig. 8a). We further investigated the NF-κB inhibitory effects in BV2 microglial and cultured astrocyte cells after LPS treatment (1 µg/mL) with MCZ pre-treatment (5 µM and 10 µM). Western blotting revealed that MCZ treatment reduced the LPS-induced nuclear translocation of the NF-κB protein, p65 and p50 in cultured astrocyte (Fig. 8b) and microglial BV2 cells (Fig. 8c) cells. Then, we identified that the suppression of IκB phosphorylation by MCZ in the two cell lines. MCZ inhibited the nuclear translocation of p50 and p65 (Fig. 8b, c). To clarify whether MCZ could influence NF-κB activity, we induced luciferase activity in cultured astrocyte and microglia BV2 cells by treatment of LPS. MCZ concentration dependently inhibited LPS-induced NF-κB transcription in both cell lines (Fig. 8d, e). We also performed immunofluorescence to detect the effect of MCZ on p65 nuclear translocation and found that MCZ pre-treatment concentration dependently prevented the LPS-induced translocation of p65 into the nucleus (Supplementary Fig. 9A, B).

## Discussion

iNOS, also known as NOS2, a member of a family of enzymes that catalyze the production of NO from l-arginine, is expressed in numerous chronic neuroinflammatory diseases^32^. In a previous study, the brain iNOS level was reported to be increased in various central nervous system diseases caused after inflammatory, infectious, or ischemic damage as well as due to brain aging^33^. Moreover, it was also reported that an increased level of iNOS in brain microvessels was detected in patients with Alzheimer’s disease (AD)^8^. Therefore, it has been suggested that iNOS could be a new biomarker and target for AD treatment^34^. In agreement with these findings, bioinformatics analysis based on big data showed that an iNOS-inhibitory compound, MCZ, could be useful in AD treatment. Based on GWAS analysis, we hypothesized that MCZ could be effective for treatment of AD. Our findings demonstrated that MCZ inhibited neuroinflammation through inhibition of iNOS expression and thus alleviated LPS-induced memory dysfunction.

Increased iNOS expression is related to various neurodegenerative diseases such as AD, Parkinson, and ischemic/reperfusion injury^32,35,36^. Expression of inflammatory proteins (iNOS and COX-2) and the production of pro-inflammatory cytokines in brain cells cause neuronal cell death^37^. In the prefrontal cortex, patients with AD showed higher iNOS protein levels than age-matched controls did^6,7^. Whereas, knock out of iNOS improved memory function in animal model^10,11^. Previous experiments showed that the LPS-treatment cognitive impairment mice model enhanced the secretion of IL-1β, IL-6, and TNF-α and the expression of iNOS and COX-2 with increased NO production^38–40^. Using this model, we found that MCZ suppressed neuroinflammation by inhibiting iNOS expression in vivo and in vitro, and thus ameliorating LPS-induced memory dysfunction. In our results, MCZ also inhibited activation of NF-κB, a transcription factor controlling iNOS expression and NF-κB regulated neuroinflammatory gene expression, such as iNOS, COX-2, GFAP, and Iba-1, and also released inflammatory cytokines, such as NO, TNF-α, IL-1β, and IL-6, in the brain and cultured microglial BV2 cells and astrocytes. Thus, the inhibition of iNOS expression by MCZ conferred a significant anti-neuroinflammatory and memory improving roles in the brain.

Evidence for the inhibitory effects of azoles on iNOS expression in mouse and human cells can be found in accumulated studies^18,19^. Other types of cells (osteoclasts) have been shown to decrease iNOS generation and inflammatory cytokines owing to the anti-inflammatory effect of MCZ^41^. To focus on neuroinflammation, there have been reports showing the neuroprotective effects of MCZ, such as remyelination and the protection of blood vessels from rupturing and inflammation in the hemorrhagic stroke model^20,21,42^. However, these data have not demonstrated the mechanisms of MCZ on iNOS expression. In our experiment, MCZ inhibited iNOS expression and activation of NF-κB, a transcriptional factor regulating iNOS. Moreover, other azole compounds such as fluconazole and clotrimazole also inhibited NO generation and iNOS luciferase activity. Indeed, other azoles also inhibit the expression of iNOS and inflammatory cytokines, such as TNF-α, and exhibit an anti-inflammatory response^43,44^. It is also noteworthy that MCZ directly bound to the iNOS monomer, which interfered with the assembly of the active dimer. We also found that MCZ binds to iNOS evidenced by pull-down assay. Our data thus indicate that MCZ could inhibit iNOS expression either by directly bind to iNOS or by inhibit NF-κB activity although more investigation should be done to clarify the action mechanisms. Our data thus suggest that suppressive effect of MCZ on iNOS could be involved with anti-inflammatory responses, and thus memory improving effects. Overall, MCZ has appropriate drug properties that could provide an effective way to control neuroinflammation and thus prevent AD progression. In addition to the antifungal activity, MCZ could be repositioning to apply drug for AD treatment.

## Supplementary information

Supplementary Figure1

Supplementary Figure2

Supplementary Figure3

Supplementary Figure4

Supplementary Figure5

Supplementary Figure6

Supplementary Figure7

Supplementary Figure8

Supplementary Figure9

Supplementary figure legends
