# Supplementary figures and images for: Antifungal drug miconazole ameliorated memory deficits in a mouse model of LPS-induced memory loss through targeting iNOS
Source: Cell Death Dis. 2020 Aug 14;11(8):623. doi: 10.1038/s41419-020-2619-5 (PMC7429861; doi:10.1038/s41419-020-2619-5)

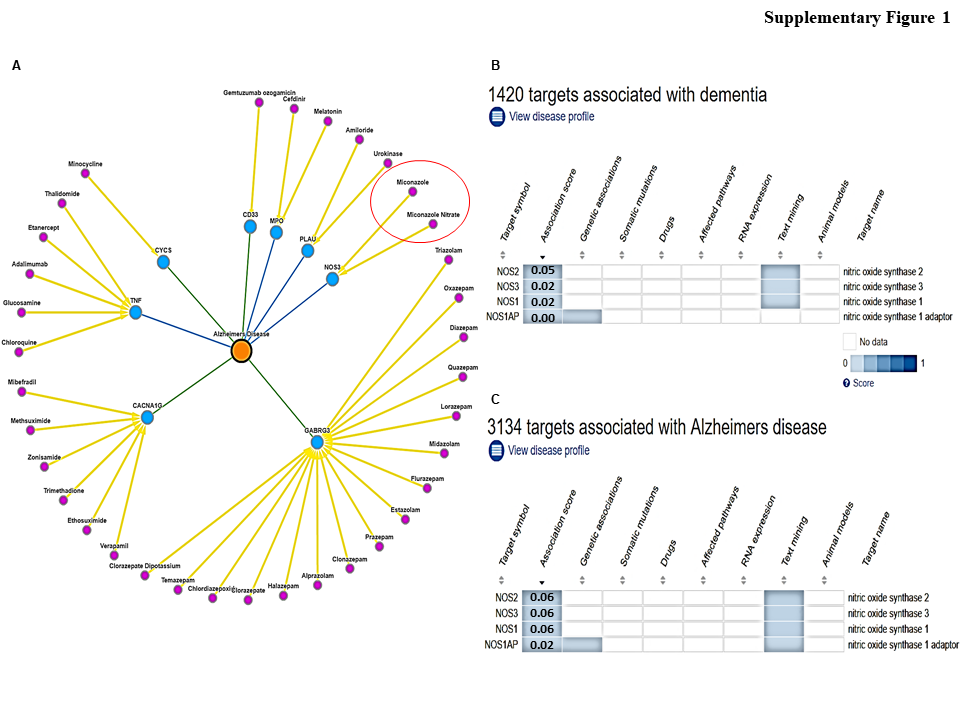

Supplement: Supplementary file 1 — Supplementary Figure1 [file 41419_2020_2619_MOESM1_ESM.tif]

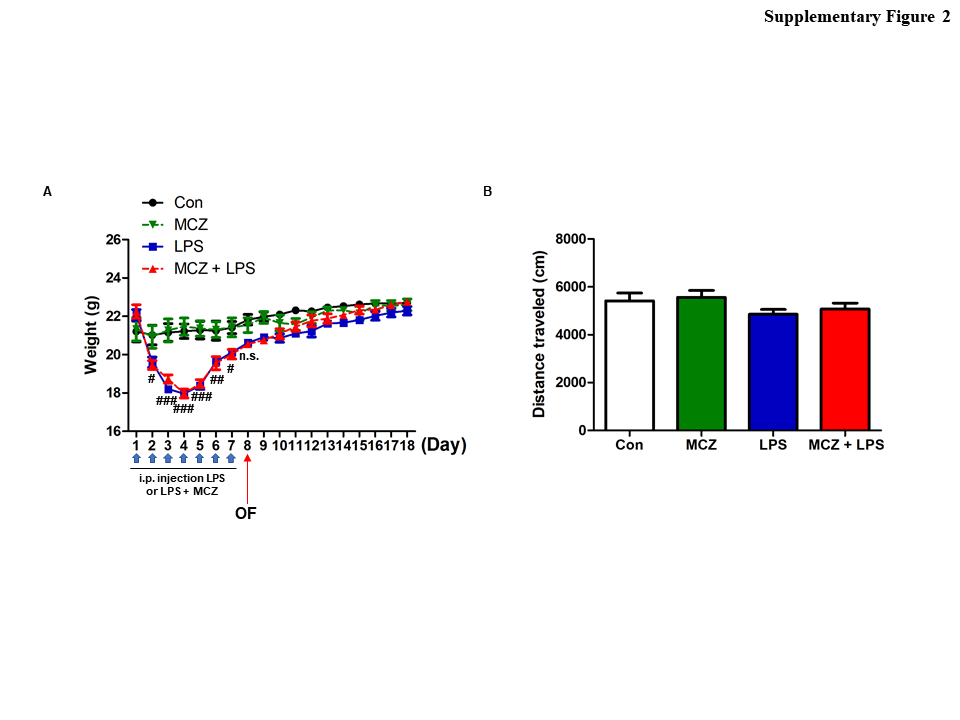

Supplement: Supplementary file 2 — Supplementary Figure2 [file 41419_2020_2619_MOESM2_ESM.tif]

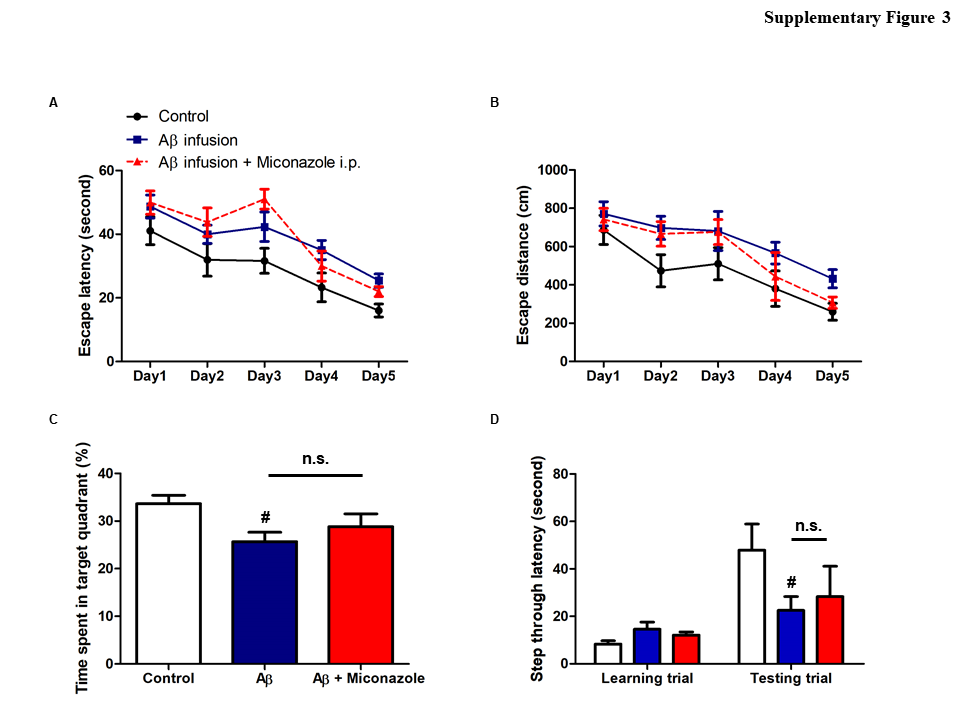

Supplement: Supplementary file 3 — Supplementary Figure3 [file 41419_2020_2619_MOESM3_ESM.tif]

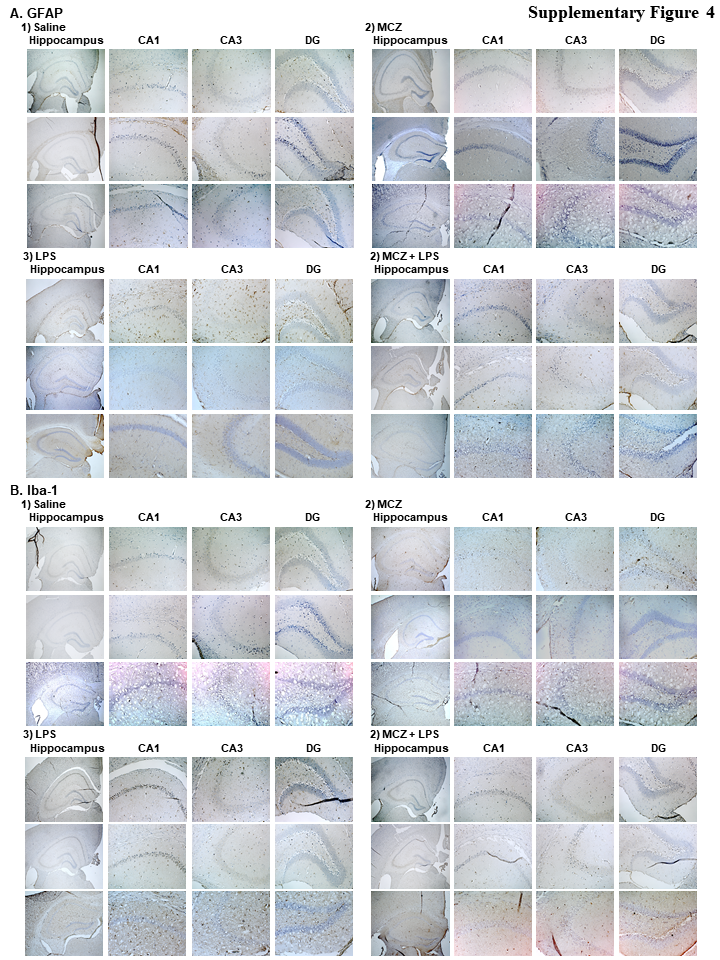

Supplement: Supplementary file 4 — Supplementary Figure4 [file 41419_2020_2619_MOESM4_ESM.tif]

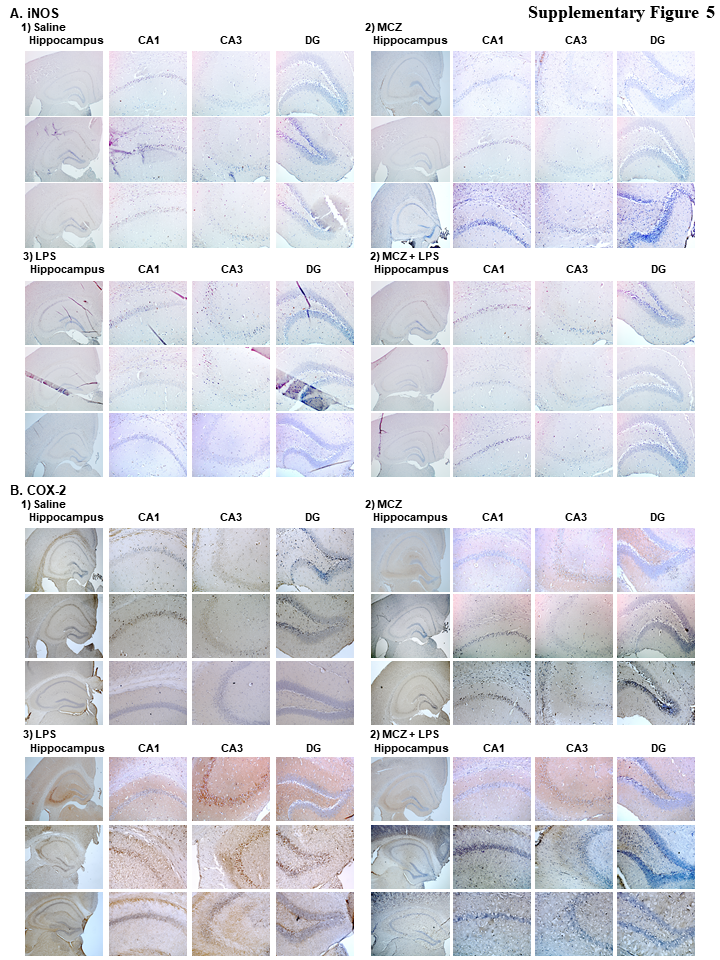

Supplement: Supplementary file 5 — Supplementary Figure5 [file 41419_2020_2619_MOESM5_ESM.tif]

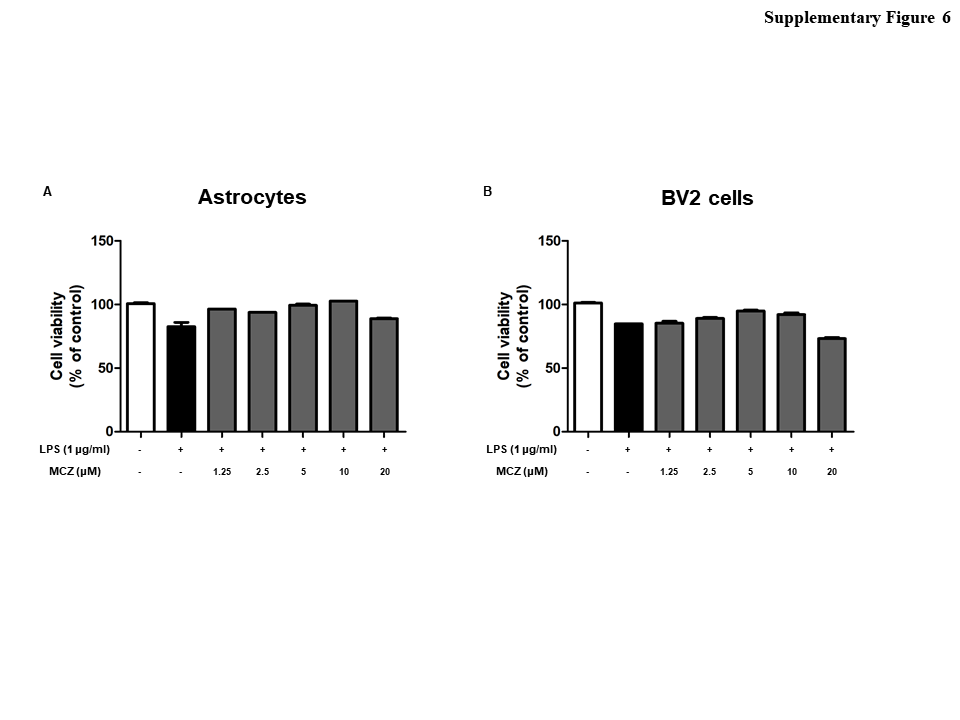

Supplement: Supplementary file 6 — Supplementary Figure6 [file 41419_2020_2619_MOESM6_ESM.tif]

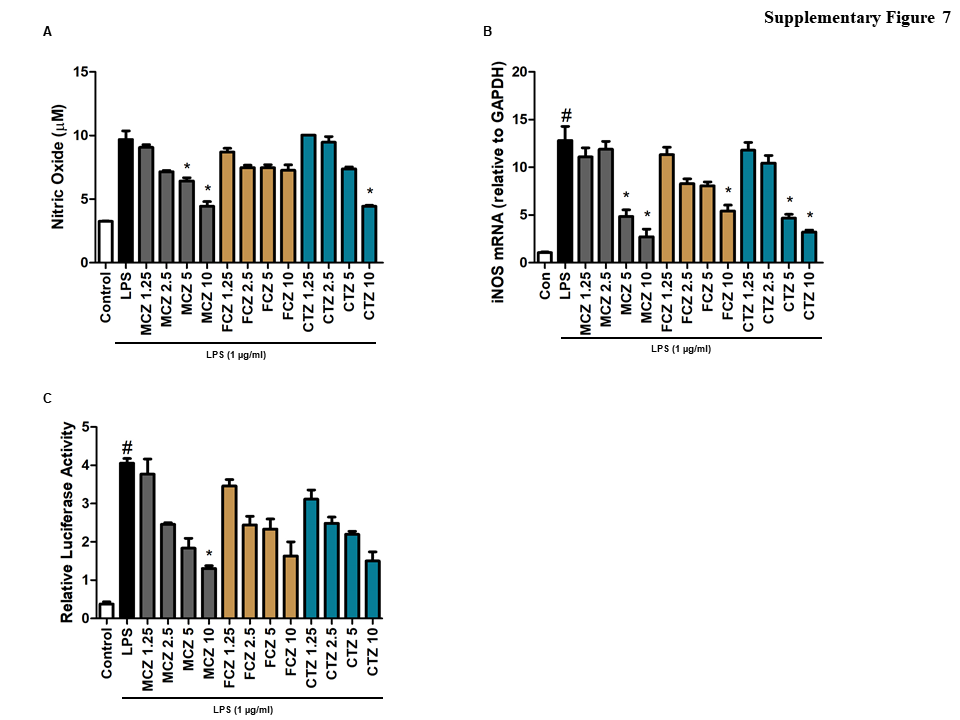

Supplement: Supplementary file 7 — Supplementary Figure7 [file 41419_2020_2619_MOESM7_ESM.tif]

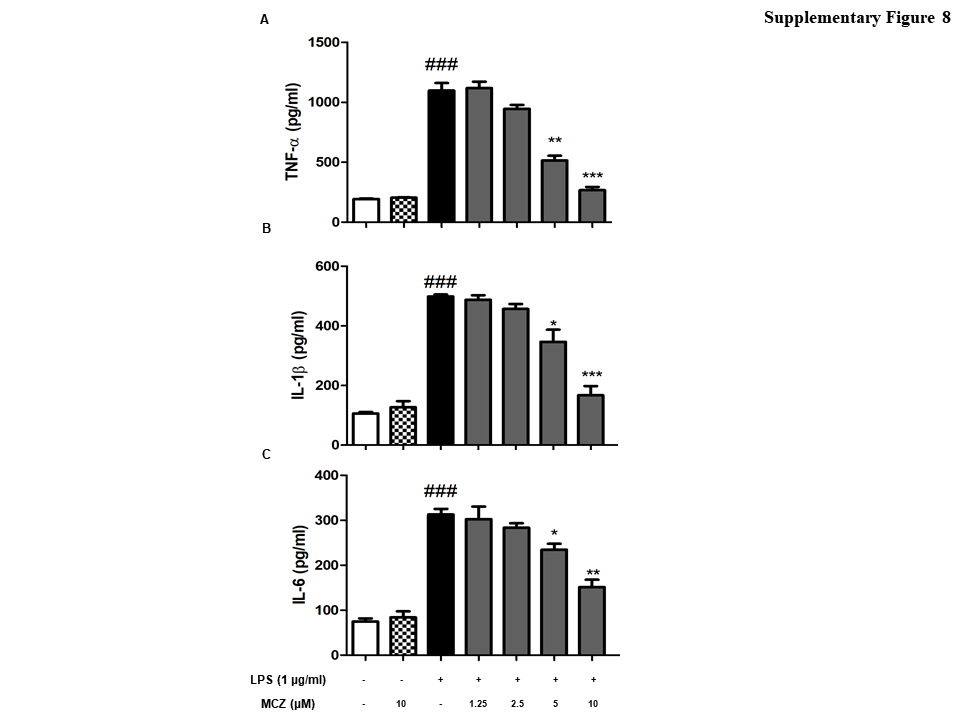

Supplement: Supplementary file 8 — Supplementary Figure8 [file 41419_2020_2619_MOESM8_ESM.tif]

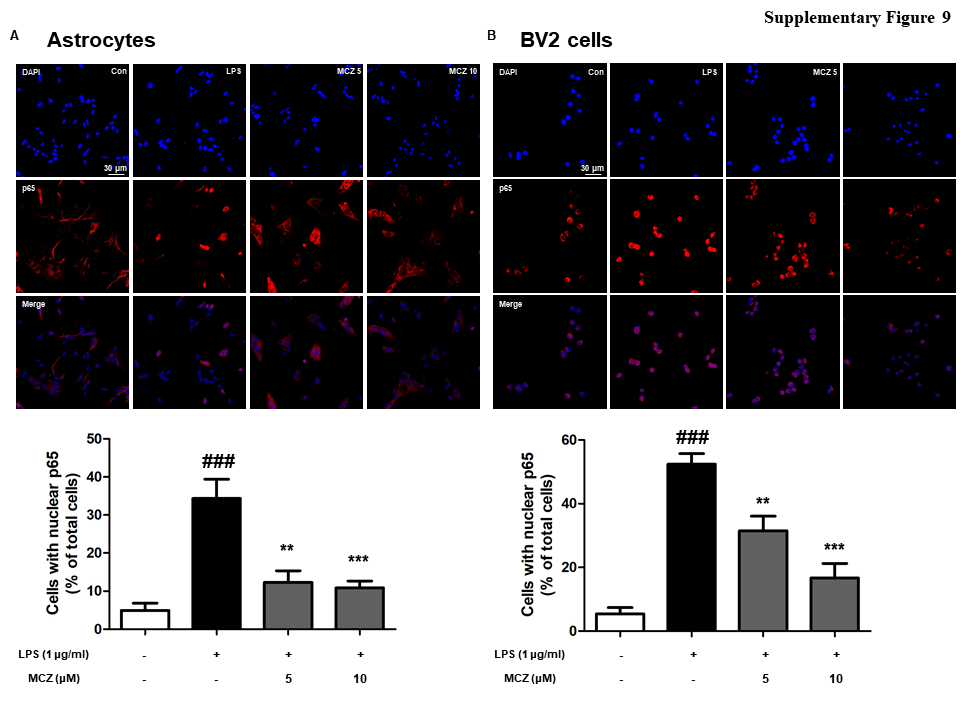

Supplement: Supplementary file 9 — Supplementary Figure9 [file 41419_2020_2619_MOESM9_ESM.tif]
