# Supplementary material for: Antifungal drug miconazole ameliorated memory deficits in a mouse model of LPS-induced memory loss through targeting iNOS
Source: Cell Death Dis. 2020 Aug 14;11(8):623. doi: 10.1038/s41419-020-2619-5 (PMC7429861; doi:10.1038/s41419-020-2619-5)
Supplement: Supplementary file 10 — Supplementary figure legends [file 41419_2020_2619_MOESM10_ESM.docx]

**Supplementary figure legends**

**Supplementary Figure 1. Disease-gene-drug network analysis based on the GWAS/OMIM/DEG records.** Drug network database with NOS and miconazole (A) using disease-connect (disease-connect.org). The relationships between NOS and Dementia (B), Alzheimer’s disease (C) using Open Target Platform ([www.targetvalidation.org](http://www.targetvalidation.org/)).

**Supplementary Figure 2. Evaluation of daily weight check and sickness behavior by open field test.** Daily weight check starts from first injection day (A). The total distance of locomotion in 30 min (n = 6 per group) (B). Data are expressed as the mean ± SEM (t test, ^#^p < 0.05, ^##^p < 0.01, ^###^p < 0.0001 vs. Con)

**Supplementary Figure 3. Behavior test with Aβ_1–42_-induced AD mice.** The Morris water maze test was performed three times a day for 5 days. Escape latency (B) and distance (C) to arrive at the platform were automatically recorded. Data are expressed as the mean ± SEM (Two-way ANOVA for repeated measures followed by post hoc Bonferroni, ^#^p < 0.05 vs. Con). After the Morris water maze test, a probe test was performed. The time spent in the target quadrant and target site crossing was within 60 s (D). Data are expressed as the mean ± SEM the passive avoidance test was performed 1 day after a learning trial test (E). The mice were given an electric shock when they entered the dark compartment. Data are expressed as the mean ± SEM (t test, ^#^p < 0.05 vs. Con).

**Supplementary Figure 4.** Immunohistochemical stainings were performed for GFAP (A) and Iba-1 (B) using frozen hippocampal tissues of saline, MCZ, LPS and MCZ + LPS treated mice (n=3 mice per group).

**Supplementary Figure 5.** Immunohistochemical stainings were performed for iNOS (A) and COX-2 (B) using frozen hippocampal tissues of saline, MCZ, LPS and MCZ + LPS treated mice (n=3 mice per group).

**Supplementary Figure 6. Cytotoxicity of miconazole on LPS-treated cultured astrocytes and microglial BV2 cells.** Cytotoxicity were measured in 24-h LPS-treated (1 μg/mL) microglial BV2 cells (A) and cultured astrocytes (B) pretreated with MCZ (1.25, 2.5, 5, 10, and 20 μM) for 1 h. The data shown represent the means ± SEM of three replicates.

**Supplementary Figure 7. Comparison of several azole compounds on NO generation and iNOS expression in LPS-treated microglial BV2 cells.** The NO generation (A), mRNA levels of iNOS (B), and iNOS luciferase activity (C) were measured in 24-h LPS-treated (1 μg/mL) microglial BV2 cells pretreated with MCZ, fluconazole, or clotrimazole for 1 h. #Significant difference from the control group (t test, p < 0.05). *Significant difference from the LPS-treated group (p < 0.05). MCZ: miconazole; FCZ: fluconazole; CTZ: clotrimazole

**Supplementary Figure 8. Cytokines secretion level effect of miconazole on LPS-treated microglial BV2 cells.** Secretion levels of cytokines were measured in 24-h LPS-treated (1 μg/mL) microglial BV2 cells pretreated with MCZ (1.25, 2.5, 5, and 10 μM) for 1 h. Supernatant was used to measure level of TNF-α (A), IL-1β (B), and IL-6 (C) by ELISA. The data shown represent the means ± SEM of three replicates. #Significant difference from the control group (t test, ^###^p < 0.0001). *Significant difference from the LPS-treated group (t test ^*^p < 0.05, ^**^p < 0.01).

**Supplementary Figure 9. Miconazole inhibits NF-κB translocation in microglial BV2 cells and cultured astrocytes.** The cultured astrocytes (A) and microglial BV2 cells (B) were incubated with anti-p65 (red) and DAPI staining (blue). Image were measured in 30-m LPS-treated (1 μg/mL) cultured astrocytes and microglial BV2 cells pretreated with MCZ (5 and 10 μM) for 1 h. The graph represents the percentage of cells with nuclear p65 measured by ImageJ #Significant difference from the control group (t test, ^###^p < 0.0001). *Significant difference from the LPS-treated group (t test ^**^p < 0.01, ^***^p < 0.0001).
